# The clinical spectrum and genetic variability of limb-girdle muscular dystrophy in a cohort of Chinese patients

**DOI:** 10.1186/s13023-018-0859-6

**Published:** 2018-08-14

**Authors:** Liang Wang, Victor Wei Zhang, Shaoyuan Li, Huan Li, Yiming Sun, Jing Li, Yuling Zhu, Ruojie He, Jinfu Lin, Cheng Zhang

**Affiliations:** 1 Department of Neurology, National Key Clinical Department and Key Discipline of Neurology, The First Affiliated Hospital, Sun Yat-sen University, 58 Zhongshan 2 Road, Guangzhou, 510080 GD China; 2 Department of Molecular and Human Genetics, Baylor College of Medicine, Houston, TX 77030 USA; 3 AmCare Genomics Lab, Guangzhou, 510300 GD China; 4 Department of Health Care, The First Affiliated Hospital, Sun Yat-sen University, Guangzhou, 510080 GD China

**Keywords:** Limb-girdle muscular dystrophy, Clinical manifestation, Muscle magnetic resonance imaging, Molecular diagnosis, South China

## Abstract

**Background:**

Limb-girdle muscular dystrophy (LGMD) is a commonly diagnosed hereditary muscular disorder, characterized by the progressive weakness of the limb-girdle muscles. Although the condition has been well-characterized, clinical and genetic heterogeneity can be observed in patients with LGMD. Here, we aimed to describe the clinical manifestations and genetic variability among a cohort of patients with LGMD in South China.

**Results:**

We analyzed the clinical information, muscle magnetic resonance imaging (MRI) findings, and genetic results obtained from 30 patients (24 families) with clinically suspected LGMD. In 24 probands, 38 variants were found in total, of which 18 were shown to be novel. Among the 30 patients, the most common subtypes were dysferlinopathy in eight (26.67%), sarcoglycanopathies in eight [26.67%; LGMD 2C in three (10.00%), LGMD 2D in three (10.00%), and LGMD 2F in two (6.67%)], LGMD 2A in seven (23.33%), followed by LGMD 1B in three (10.00%), LGMD 2I in three (10.00%), and early onset recessive Emery-Dreifuss-like phenotype without cardiomyopathy in one (3.33%). Furthermore, we also observed novel clinical presentations for LGMD 1B, 2F, and 2I patients with hypermobility of the joints in the upper limbs, a LGMD 2F patient with delayed language development, and other manifestations. Moreover, distinct distributions of fatty infiltration in patients with LGMD 2A, dysferlinopathy, and the early onset recessive Emery-Dreifuss-like phenotype without cardiomyopathy were also observed based on muscle MRI results.

**Conclusions:**

In this study, we expanded the clinical spectrum and genetic variability found in patients with LGMD, which provided additional insights into genotype and phenotype correlations in this disease.

**Electronic supplementary material:**

The online version of this article (10.1186/s13023-018-0859-6) contains supplementary material, which is available to authorized users.

## Background

Limb-girdle muscular dystrophy (LGMD) is a commonly diagnosed hereditary muscular disorder, characterized by progressive weakness of the limb-girdle muscles [1, 2]. As a group of neurogenetic diseases, LGMD is the fourth most common muscular dystrophy, with a pooled prevalence of 1.63 per 100,000 people, following myotonic dystrophy, dystrophinopathy, and facioscapulohumeral dystrophy [1–4]. The prevalence varies among subtypes and regions. For example, the most common subtypes in Italy are LGMD 2A and 2B, while LGMD 2I is most common in Denmark [5, 6]. LGMD was first described by Erb and Leyden-Möbius in the late eighteenth century, and 30 distinct subtypes have been identified thus far [7]. On the basis of the origin of muscle weakness, LGMD was initially divided into the Erb phenotype (scapulohumeral type), Leyden-Möbius phenotype (pelvic girdle type), and Miyoshi phenotype (exceptionally distal type) [8]. Later, classifications were made based on the mode of inheritance; thus, LGMD patients can be subdivided into two main classes: LGMD subtype 1 with autosomal dominant inheritance and LGMD subtype 2 with autosomal recessive inheritance [9]. LGMD subtype 2 (2A-2 U) is more common than LGMD subtype 1 (1A-1H), and most of these subtypes have been shown to be associated with defects in specific genes [7].

LGMD is mainly described as progressive weakness and atrophy of the hip, shoulder, and proximal extremity muscles, with onset in the second decade of life [9]. Considering the genetic and phenotypic heterogeneity of this disease, LGMD should be considered in almost all undiagnosed patients complaining of primary muscle weakness [10]. Certain clinical features of LGMD subtypes can serve as valuable diagnostic clues, such as extremely high levels of serum creatine kinase (CK) in LGMD 2B-F and LGMD 2I patients [11]. Other approaches such as muscle biopsy and muscle magnetic resonance imaging (MRI) may also help establish the clinical diagnosis [12, 13]. Moreover, genetic testing can provide accurate information for unambiguously establishing the genetic diagnosis, and it is currently available at a very low cost owing to the rapid development of sequencing techniques [14, 15]. Therefore, a comprehensive understanding of the genetic variability and clinical spectrum of LGMD is essential before precise diagnosis, treatment and reproductive counselling can be offered [2].

Previously, clinical characteristics and the underlying molecular defects have been documented in patients with LGMD [5, 16, 17]. Here, we aimed to extend our knowledge and understanding of this disease to determine novel and important characteristics and genetic variants in patients with LGMD.

## Methods

### Participants and patient information

Thirty patients suspected to have LGMD were enrolled in this study from 2015 to 2017 from the Hereditary Neurological Disease Clinics of The First Affiliated Hospital, Sun Yat-sen University. The basic data obtained from these patients were recorded, including clinical histories and motor functions, and were evaluated by two experienced neurologists. Ages are presented as mean ± standard deviation. The activity of CK was examined in each patient. Furthermore, we performed electromyography (EMG), cardiac function estimation, muscle tissue histology, immunohistochemical staining (IHC), and MRI according to previously published protocols [18, 19].

### Muscle histology

Muscle biopsy specimens were obtained from the biceps brachii or quadriceps femoris. Biopsied muscles were frozen in isopentane chilled by liquid nitrogen. IHC staining was performed on 6 μm frozen muscle sections according to the established protocol [20]. Antibodies against dystrophin, α-sarcoglycan, β-sarcoglycan, γ-sarcoglycan, and δ-sarcoglycan were purchased from Novocastra (Newcastle, UK), while the anti-dysferlin antibody was purchased from Millipore (Darmstadt, Germany).

### Next-generation sequencing

Blood samples (2 mL) from each patient were collected in EDTA-coated tubes. DNA (3 μg) was extracted from these samples using the QIAamp DNA Blood Midi kit (Qiagen, Hilden, Germany). A next-generation sequencing (NGS) neuromuscular disorder panel was used to detect variants. The detailed NGS protocol and the list of genes detected using this panel are provided in Additional file 1. Briefly, the process included the construction of a genomic DNA library, capturing the locations of target genes, gene sequencing using an analytical platform (HiSeq 2000 system; Illumina, USA), and bioinformatics analysis. After the identification of the variants in the proband, family co-segregation analyses were performed using Sanger sequencing.

### Bioinformatics analysis

Gene frequencies were determined using the 1000 Genomes Project and ExAC [21, 22], and computational analysis of variants was performed using PolyPhen-2, SIFT, and MutationTaster [23–25]. The Human Gene Mutation Database, Leiden Open Variation Database, ClinVar, and Google Scholar were used to identify the reported variants [26–29]. The pathogenicity of the variants was estimated using the American College of Medical Genetics and Genomics (ACMG) guidelines [30].

## Results

### Participants

Detailed data were collected from 24 probands (Table 1). There were, in total, 30 patients in 24 families. Proband ages were between 3 and 44 years. There were 14 female probands [58.33% of probands; 18 female patients (60.00% of patients)] and 10 male probands [41.67% of probands; 12 male patients (40.00% of patients)]. Autosomal recessive types (LGMD 2) were most common [21 probands (87.50% of probands) and 27 patients (90.00% of patients)].

**Table 1:** Clinical data of probands

Proband/Gender	Age of last review/onset (y)	Phenotype	Pathogenic gene	Motor function/muscle weakness	Hypertrophy of calves	Tendon reflex	Join contracture	CK (U/L)	EMG	Other
1/M	44/18	LGMD 2A	*CAPN3*	Nonambulant at 40y; difficulty in raising arms at 22y; UL: proximal; LL: proximal	No	–	NA	424	Myopathic	Hypertension in all patients of the family
2/F	23/17	LGMD 2A?	*CAPN3?*	Ambulant; LL: proximal, mild distal	No	+	Ankle	2375	Myopathic	Lordosis
3/M	29/19	Dysferlinopathy	*DYSF*	Ambulant; LL: mild proximal, distal	No	+	Ankle	4032	Myopathic	
4/F	9/8	LGMD 2C	*SGCG*	Ambulant; LL: proximal, mild distal	Present	+	No	15,025	NA	
5/F	34/21	Dysferlinopathy	*DYSF*	Ambulant; LL: proximal, distal	No	+	No	4777	Myopathic	Lordosis
6/F	24/16	Dysferlinopathy	*DYSF*	Ambulant; LL: proximal, distal	No	+	Ankle	8720	Myopathic	
7/M	30/8	LGMD 2C	*SGCG*	Nonambulant at 21y; difficulty in raising arms at 22y; UL: proximal; LL: proximal	No	–	No	3200	NA	
8/F	37/29	Dysferlinopathy	*DYSF*	Ambulant; difficulty in raising arms at 35y; UL: proximal; LL: proximal, distal	No	–	No	6606	Myopathic	
9/F	25/15	LGMD 2A	*CAPN3*	Ambulant; difficulty in raising arms at 25y; UL: proximal; LL: proximal, distal	No	–	Ankle	2819	NA	Lordosis
10/F	12/10	LGMD 2D	*SGCA*	Ambulant; difficulty in raising arms at 11y; UL: proximal; LL: proximal	No	+	NA	17,690	NA	Lordosis
11/M	11/6	LGMD 2I	*FKRP*	Ambulant; LL: mild proximal	Mild	+	No	8610	NA	Hypermobility of metacarpophalangeal joints
12/M	12/6	Early onset recessive Emery-Dreifuss-like phenotype without cardiomyopathy	*TTN*	Ambulant; UL: mild proximal; LL: mild proximal, distal	No	+	Neck, ankle	378	Normal	Cavus feet, Morton’s toe, tongue hypertrophy, winged shoulder
13/M	21/16	Dysferlinopathy	*DYSF*	Ambulant; LL: mild proximal, distal	No	+	No	17,139	Myopathic	
14/F	22/20	Dysferlinopathy	*DYSF*	Ambulant; LL: mild proximal	No	+	No	8179	Myopathic	Six fingers in both hands
15/F	8/3	LGMD 2D	*SGCA*	Ambulant; LL: proximal, mild distal	No	+	No	58,378	Myopathic	
16/M	13/13	Dysferlinopathy	*DYSF*	Ambulant; LL: mild proximal, mild distal	No	++	No	32,093	Normal	Patent foramen ovale
17/F	14/3	LGMD 2F	*SGCD*	Nonambulant at 12y; difficulty in raising arms at 13y; UL: proximal; LL: proximal, mild distal	Present	–	Ankle	1748	Myopathic	Hypermobility of interphalangeal, metacarpophalangeal and elbow joints
18/F	7/2	LGMD 1B	*LMNA*	Ambulant; LL: proximal	No	–	Ankle	1806	Myopathic	Hypermobility of interphalangeal and metacarpophalangeal joints
19/M	3/1.5	LGMD 1B	*LMNA*	Ambulant; LL: proximal	Mild	+	No	1406	NA	Lordosis
20/F	3/2	LGMD 1B	*LMNA*	Ambulant; LL: proximal	No	+	Ankle	1311	Myopathic	Lordosis
21/F	35/25	LGMD 2I	*FKRP*	Ambulant; UL: mild proximal; LL: proximal, mild distal	NA	+	No	420	Myopathic	Lordosis
22/M	23/13	LGMD 2C	*SGCG*	Nonambulant at 22y; difficulty in raising arms at 20y; UL: proximal; LL: proximal	Mild	–	No	2700-5000	Myopathic	
23/M	7/2.5	LGMD 2F	*SGCD*	Ambulant; LL: proximal, mild distal	Present	+	No	18,915	NA	Delayed language development
24/F	28/13	LGMD 2A	*CAPN*	Ambulant; difficulty in raising arms; UL: proximal; LL: proximal, mild distal	Mild	NA	NA	NA	Myopathic	Lordosis

*M* male, *F* female, *UL* upper limbs, *LL* lower limbs, *NA* not available, *EMG* electromyography, *++* normal tendon reflex, *+* decreased tendon reflex, – absent tendon reflex

The most frequent subtypes were dysferlinopathy [seven probands (29.17% of probands), eight patients (26.67% of patients)], sarcoglycanopathies [LGMD 2C-F; seven probands (29.17% of probands), eight patients (26.67% of patients)], LGMD 2A [four probands (16.67% of probands), seven patients (23.33% of patients)], and LGMD 1B [three probands (12.50% of probands); three patients (10.00% of patients)]. Within sarcoglycanopathies, the frequencies of LGMD 2C, 2D, and 2F were three probands [12.50% of probands; three patients (10.00% of patients)], two probands [8.33% of probands; three patients (10.00% of patients)], and two probands [8.33% of probands; two patients (6.67% of patients)], respectively. The remaining patients were affected by LGMD 2I [two probands (8.33% of probands), three patients (10.00% of patients)], except for a patient with the early onset recessive Emery-Dreifuss-like phenotype without cardiomyopathy. Because the disease history of the proband in each family could be collected accurately, the main results in this study represent the data of 24 probands, as below.

### Age of onset

The subtype with the earliest age of onset was LGMD 1B (1.83 ± 0.29 years), followed by LGMD 2F (2.5 and 3 years). Overall, the age of onset of sarcoglycanopathies was early, since most of them presented with muscle weakness no later than 10 years (6.79 ± 4.06 years). In addition, most probands with LGMD 2A and dysferlinopathy presented with muscle weakness during the second decade of life, wherein the ages of onset in LGMD 2A and dysferlinopathy were 15.75 ± 2.22 years and 19.14 ± 5.15 years, respectively. Proband 12 had the early onset recessive Emery-Dreifuss-like phenotype without cardiomyopathy and complained of muscle weakness since the age of 6 years. Moreover, the difference of age of onset between two probands with LGMD 2I was large (6 and 25 years).

### Muscle weakness and motor function

All probands had weakness of the proximal lower limbs to different degrees. In 10 probands (41.67%), the proximal upper limbs were involved, among whom nine probands (37.50%) had predominant weakness of both proximal upper limbs and proximal lower limbs. Furthermore, 15 probands (62.50%) complained of weakness of the distal lower limbs, among whom eight probands had mild symptoms. When analyzing predominant involvements of proximal or distal limbs, the results indicated that most probands were characterized by predominant weakness of the scapular and/or pelvic muscles (83.33%); however, three probands (proband 3 and 13 with dysferlinopathy and proband 12 with the early onset recessive Emery-Dreifuss-like phenotype without cardiomyopathy) were characterized by predominant weakness of the distal lower limbs and one proband, with dysferlinopathy (proband 16) characterized by mild weakness of both proximal and distal lower limbs.

Four probands (16.67%) lost the ability of ambulation, among whom three had sarcoglycanopathies and one had LGMD 2A. Of 20 probands with the ability of ambulation, eight presented with an obvious waddling gait. When we evaluated probands’ daily movements, we found that the number of probands losing the ability of jumping, climbing, and standing up from the ground were 10 (LGMD 2A: two; dysferlinopathy: three; sarcoglycanopathies: two; LGMD 1B: two; LGMD 2I: one), one (dysferlinopathy: one), and four (LGMD 2A, dysferlinopathy, LGMD 2D, and LGMD 2I, one each), respectively, in the probands with the ability of ambulation. Furthermore, four probands could jump normally (dysferlinopathy: two; LGMD 2I: one; early onset recessive Emery-Dreifuss-like phenotype without cardiomyopathy: one). A proband with dysferlinopathy could climb normally, and two probands with dysferlinopathy could stand up from the ground normally. The remaining probands had difficulties in these movements to different degrees. In addition, eight probands (33.33%) had difficulties in raising arms, among whom four, three, and one had sarcoglycanopathies, LGMD 2A, and dysferlinopathy, respectively.

### Disease course

All probands presented with progressive deterioration of muscle weakness. The mean time between disease onset and last review was 7.79 ± 6.43 years. Among four probands losing ambulation, the mean time between loss of ambulation and onset of disease for patients with sarcoglycanopathies was 10.00 years, and proband 1 with LGMD 2A lost the ability of ambulation at the age of 40 years, i.e., 22 years after the onset of weakness. The time between difficulty in raising arms and onset of disease for probands with sarcoglycanopathies varied from 1 year to 14 years (mean, 8 years), and the time for probands 1 and 9 with LGMD 2A and proband 8 with dysferlinopathy were 4, 10, and 6 years, respectively. The onset age of raising arms with difficulty in proband 24 with LGMD 2A was not available.

### Rare clinical features

**Fig. 1:**
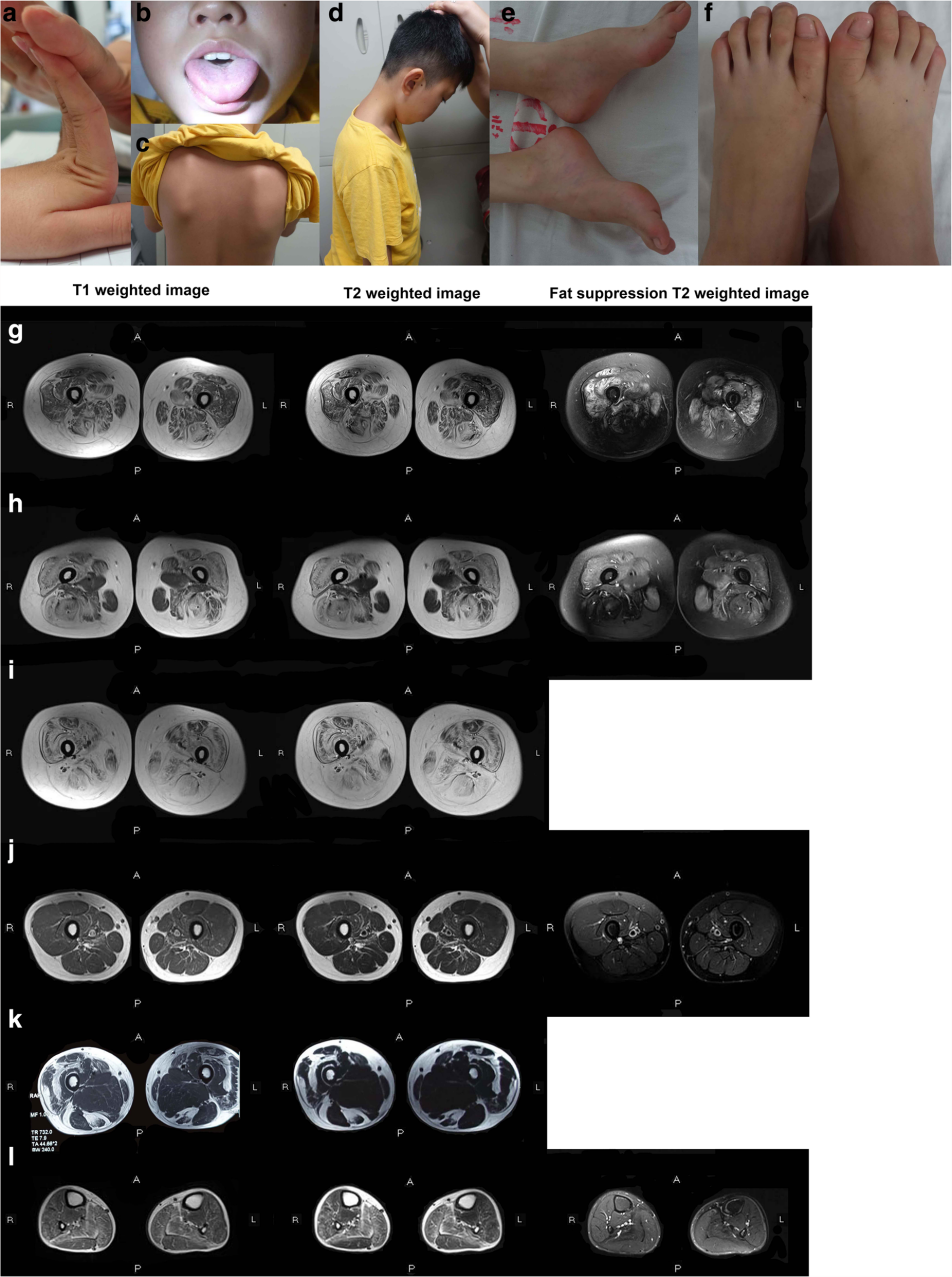
Clinical and magnetic resonance imaging (MRI) results obtained for all patients included in the study. (**a**) Hypermobility of the metacarpophalangeal joints observed in proband 11. (**b**–**e**) Physical characteristics of proband 12. (**b**) Tongue hypertrophy. (**c**) Winged shoulder. (**d**) Neck contracture. (**e**) Ankle contracture and cavus feet. (**f**) Morton’s toe. (**g**–**l**) Muscle MRI results obtained for probands 3, 5, 9, 11, and 12. T1-weighted image, T2-weighted image, and fat-suppression T2-weighted image (left to right). MRI results of both thighs obtained for proband 3 (**g**), proband 5 (**h**), proband 9 (**i**), proband 11 (**j**), and proband 12 (**k**). (**l**) MRI results of both calves obtained for proband 12

In this cohort, three probands had hypermobility of the joints. Proband 11 with LGMD 2I presented with hypermobility of the metacarpophalangeal joints (Fig. 1a), and proband 17 with LGMD 2F presented with hypermobility of the interphalangeal, metacarpophalangeal, and elbow joints. Furthermore, proband 18 with LGMD 1B presented with hypermobility of the interphalangeal and metacarpophalangeal joints.

In addition, proband 14 with dysferlinopathy had six fingers in both hands and proband 16 with dysferlinopathy had patent foramen ovale in his heart. Proband 23 with LGMD 2F also demonstrated delayed language development. He could speak only three to five simple words at the age of 3 years, and language learning was slower compared to normal children. Language ability ameliorated gradually, and he could understand orders and have a conversation with other people at the age of 7 years.

Proband 12 presented a rare phenotype of early onset recessive Emery-Dreifuss-like phenotype without cardiomyopathy; thus, we describe his disease history in detail. He complained of mild difficulties when climbing and standing up from the ground since the age of 6 years, and muscle weakness progressed slowly with time. He was admitted to our clinic at the age of 12 years, with mild muscle weakness. Compared with that observed in the proximal limbs, dorsiflexion of the ankle muscles was obviously weaker, and he could not complete the sit-up movement. Facial, bulbar, and oculomotor muscles were not affected. Tongue hypertrophy (Fig. 1b) and winged shoulder (Fig. 1c) could be observed. His neck could not flex normally (Fig. 1d) and ankle could not dorsiflex fully (Fig. 1e) because of contractures. Moreover, some malformations of the feet were observed, such as cavus feet (Fig. 1e) and Morton’s toe (the second toe was longer than the big toe, Fig. 1f) in both feet.

### CK levels, EMG, muscle histology, and cardiac examinations

In 23 probands who underwent serum enzyme tests, CK levels (reference range: 25–200 U/L) were elevated. For the majority (69.57%), the levels reached more than tenfold over the upper normal limit (ULN). Among probands (26.09%) whose CK levels were more than 50-fold higher than the ULN, four demonstrated sarcoglycanopathies and two, dysferlinopathy. Furthermore, the CK levels of all probands with LGMD 1B varied between fivefold and tenfold over the ULN. Among three probands whose CK levels were less than fivefold higher than the ULN, one each had advanced LGMD 2A (proband 1), the early onset recessive Emery-Dreifuss-like phenotype without cardiomyopathy (proband 12), and LGMD 2I (proband 21). Furthermore, all probands who underwent EMG except for probands 12 and 16 exhibited myopathic injuries (Table 1).

Nine probands underwent cardiac examinations. The cardiac examination results, including electrocardiography and ultrasonic cardiography, of probands 3, 11, 12, 15, 18, and 21 were within normal limits. The remaining results indicated cardiac injuries. Mild mitral regurgitation was observed in proband 14, and patent foramen ovale, mild left atrial and ventricular enlargement were observed in proband 16. Besides, left ventricular high voltage was observed in proband 17.

Eight probands (probands 2, 3, 10, 11, 13, 14, 23, and 24) underwent muscle biopsy and histology analysis. Typical muscular dystrophy phenotypes were observed in all patients, including degeneration, regeneration, centralized myofiber nuclei, and different levels of connective tissue hyperplasia. Among these eight probands, muscle sections of probands 2 and 10 were stained with antibodies against dystrophin, dysferlin, α-sarcoglycan, β-sarcoglycan, γ-sarcoglycan, and δ-sarcoglycan. Although the muscle sections of proband 2 were not found to have any negative staining, IHC analysis results obtained for proband 10 were negative for sarcoglycan-α and sarcoglycan-β staining, indicating sarcoglycanopathies. Muscle sections of probands 11, 13, and 23 were only stained with anti-dystrophin, and the results indicated positive staining. The remaining muscle samples were not stained using IHC.

### Muscle MRI

MRI scans of the thigh were performed in probands 3, 5, 9, 10, 11, 12, and 15, and calf scans were performed in probands 12 and 13. The scan results of proband 3 (Fig. 1g) indicated fatty infiltration to various degrees in each muscle of both thighs, particularly in the adductor longus and posterior group of muscles (semitendinosus, semimembranosus, and biceps femoris). The involvement of the gracilis, sartorius, and quadriceps femoris was milder. Interestingly, the analysis of muscle edema indicated an opposite feature in the distribution of the involved muscles. The edema of the adductor longus and posterior group of muscles was milder than that of other muscles.

Obvious fatty infiltration of the thigh muscles was also observed in proband 5 (Fig. 1h); however, the adductor longus and gracilis were spared. Furthermore, fatty infiltration of the adductor magnus was relatively milder. In comparison to selective involvement of fatty infiltration, edema was more extensive in the thigh muscles. Similarly, fatty infiltration of the thigh muscles was also extensive in proband 9 (Fig. 1i). Obvious fatty infiltration was exhibited in almost all muscles. The involvement of the rectus femoris was relatively milder.

The sartorius, gracilis, and semitendinosus were spared in proband 10, and other muscles of the thighs represented different degrees of fatty infiltration. As for edema condition, the majority of the thigh muscles represented edema, except the sartorius, gracilis, semitendinosus, and adductor longus. In contrast, the fatty infiltration and edema of the thigh muscles were very mild in proband 11 (Fig. 1j). Mild patch-like fatty infiltration could be seen only in the adductor magnus.

The selective involvement of fatty infiltration in proband 12 was noteworthy. In the thigh muscles (Fig. 1k), the following muscles were spared from fatty infiltration: the adductor magnus, adductor longus, gracilis, vastus medialis, vastus internus, biceps femoris, and semimembranosus. Mild involvements were observed in the sartorius and rectus femoris. Severe fatty infiltration was observed in the semitendinosus and vastus lateralis. In the calf muscles (Fig. 1l), extensive fatty infiltration was found; however, the involvement of the posterior group of muscles (gastrocnemius and soleus) was more severe than that of the other muscles. This selective involvement was not observed in the edema of the calf muscles because edema with similar degrees was observed in each muscle of the calves.

Mild fatty infiltration of the calf muscles in proband 13 was found in the flexor digitorum longus and posterior group of muscles. Furthermore, muscle edema was also mild, which could be observed in the tibialis posterior, peroneus longus, and flexor digitorum longus. Furthermore, different degrees of fatty infiltration were observed in the thigh muscles of proband 15; however, the semitendinosus, sartorius, and gracilis were spared. Extensive edema of the thigh muscles was also observed in proband 15.

### Molecular analyses

NGS analyses were performed using the samples obtained from all probands to establish a precise molecular diagnosis. After the screening of DNA sequence variants according to published protocols [14], a total of 38 genetic variants were identified, including those in the *DYSF* (13 variants), *CAPN3* (seven variants), *SGCG* (four variants), *SGCA* (four variants), *FKRP* (three variants), *LMNA* (three variants), *SGCD* (two variants), and *TTN* (two variants). Compound heterozygous variants were most common (15 of 24 probands, 62.50%), followed by homozygous variants (6 of 24 probands, 25.00%) and heterozygous variants (3 of 24 probands, 12.50%). Heterozygous variants were only observed in three probands with LGMD 1B.

#### Novel variants

Eighteen of these variants were shown to be novel: c.714G > T (p.K238 N), c.5909C > T (p.P1970L), c.5357G > A (p.W1786*), and c.3550C > T (p.Q1184*) in *DYSF*; c.468C > A (p.I156=), c.382G > T (p.D128Y), and c.753delC (p.I251Ifs*2) in *CAPN3*; c.702 + 1G > A, c.756G > A (p.W252*), and c.477delG (p.V160Lfs*8) in *SGCG*; c.892delC (p.L298Cfs*23) and c.262delT (p.F88Sfs*123) in *SGCA*; c.1336G > T (p.D446Y) and c.431A > C (p.K144 T) in *LMNA*; c.503-2A > G and c.414delA (p.K138Nfs*4) in *SGCD*; and c.25006 T > C (p.C8336R) and c.33938dupC (p.E11314Rfs*24) in *TTN*, while other identified variants have been reported earlier (Table 2) [31–43]. In family 6, in addition to two pathogenic variants, c.565C > G (p.L189 V) and c.4742G > A (p.R1581H) in *DYSF* were found to be single nucleotide polymorphisms (SNPs). The pathogenicity of the unreported synonymous variant in *CAPN3*, detected in proband 2, remains uncertain. Variants in genes related with Emery–Dreifuss muscular dystrophy (*EMD*, *LMNA*, *SYNE1*, *SYNE2*, and *FHL1*) were not found in proband 12.

**Table 2:** Genetic variants detected in the patients

Mutant gene	Transcript ID	Family ID	Variant (Paternal)	Variant (Maternal)	Variant (De novo)
*DYSF*	NM_003494	Family 3	c.863A > T (p.D288V) in homozygous state		
		Family 5	c.799_800delTT (p.F267Lfs*5)	c.680 T > C (p.I227T) / **c.714G > T (p.K238N)**	
		Family 6	c.5975delT (p.V1992Efs*20)	**c.5909C > T (p.P1970L)**	
		Family 8	c.1375dupA (p.M459Nfs*15)	c.4200delC (p.I1401Sfs*47)	
		Family 13	c.3988C > T (p.Q1330*)	c.1667 T > C (p.L556P)	
		Family 14	c.1555G > A (p.G519R)	c.5357G > A (p.W1786*)	
		Family 16	c.799_800delTT (p.F267Lfs*5)	c.3550C > T (p.Q1184*)	
*CAPN3*	NM_000070	Family 1	c.1971_1973del (p.F658del) in homozygous state^b^		
		Family 2	c.439C > T (p.R147*)	**c.468C > A (p.I156=)** ^**a**^	
		Family 9	c.1795dupA (p.T599Nfs*33)	c.2050 + 1G > A	
		Family 24	**c.382G > T (p.D128Y)**	c.753delC (p.I251Ifs*2)	
*LMNA*	NM_170707	Family 18			**c.1336G > T (p.D446Y)**
		Family 19			c.1357C > T (p.R453W)
		Family 20			**c.431A > C (p.K144T)**
*SGCG*	NM_000231	Family 4	c.768delC (p.S257Afs*23) in homozygous state		
		Family 7	**c.702 + 1G > A in homozygous state**		
		Family 22		c.756G > A (p.W252*)	c.477delG (p.V160Lfs*8)
*SGCA*	NM_000023	Family 10	c.292C > T (p.R98C)	c.892delC (p.L298Cfs*23)	
		Family 15	c.262delT (p.F88Sfs*123)	c.409G > A (p.E137K)	
*SGCD*	NM_000337	Family 17	**c.503-2A > G in homozygous state**		
		Family 23	c.414delA (p.K138Nfs*4) in homozygous state		
*FKRP*	NM_001039885	Family 11	c.948delC (p.C317Afs*111)	c.545A > G (p.Y182C)	
		Family 21	compound heterozygous variant [c.151G > T (p.V51F), c.545A > G (p.Y182C)]^c^		
*TTN*	NM_001267550	Family 12	**c.25006 T > C (p.C8336R)**	c.33938dupC (p.E11314Rfs*24)	

Bold variants are novel; the symbol * means the occurence of premature temination codon; the pathogenicity of variant^a^ is uncertain

Homozygous variant^b^ was found in proband, and the proband’s mother was a carrier. But proband’s late father was undetermined

Compound heterozygous variant^c^ was found in proband, and the proband’s daughter was a carrier (c.545A > G). But proband’s parents refused to receive a genetic testing

#### Familial cosegregation analyses

**Fig. 2:**
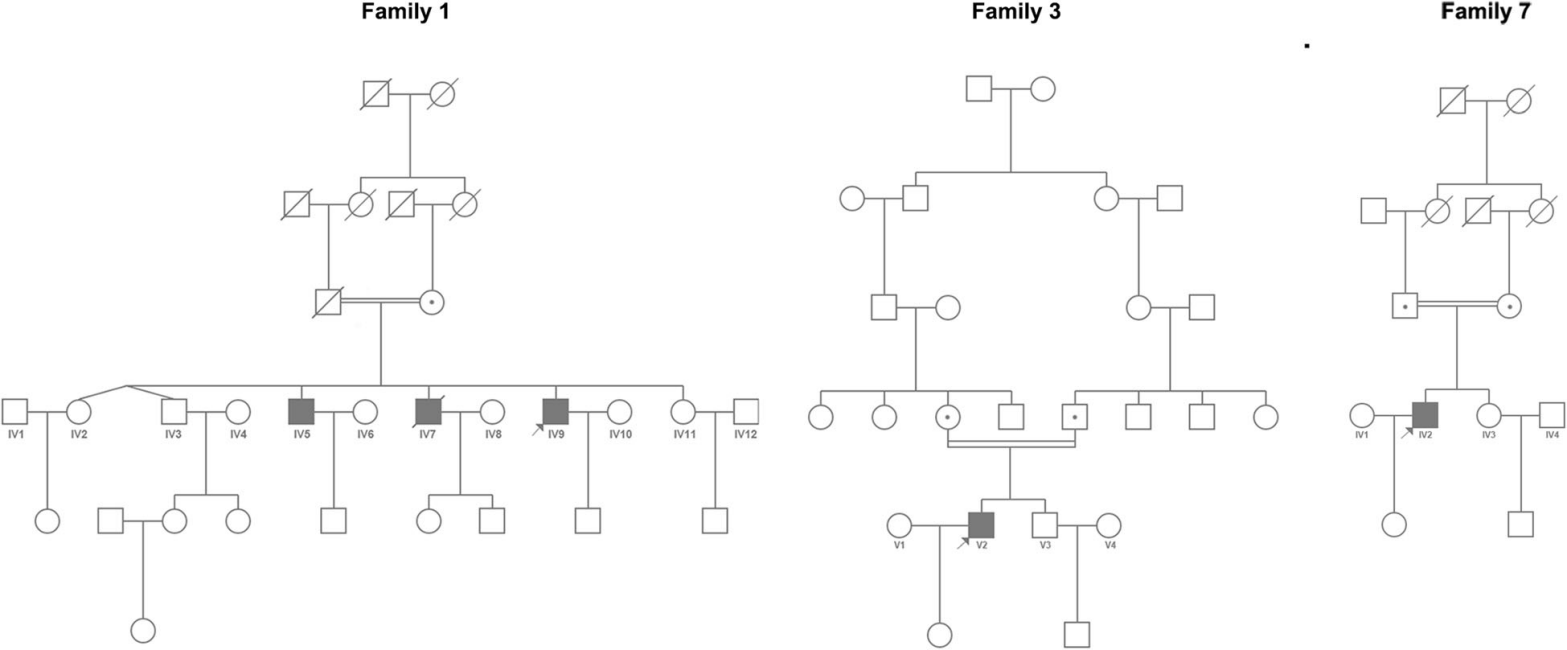
Pedigrees of families with consanguineous marriages

The blood samples collected from probands’ parents were analyzed for familial cosegregation. Most familial cosegregations were verified, except for families 1 and 21, owing to the death of the father and parents’ refusal to offer samples, respectively. A homozygous variant c.1971_1973del (p.F658del) in *CAPN3* was found in proband 1 with consanguineous parents. His mother was identified as a carrier; his late father is a suspected carrier; and the proband’s brother (IV5; Fig. 2) was shown to have the same disease and to harbor the same homozygous variant. Furthermore, variants of proband 21 [c.151G > T (p.V51F), c.545A > G (p.Y182C)] were compound heterozygous, because her daughter was a carrier of the variant c.545A > G but not c.151G > T.

**Fig. 3:**
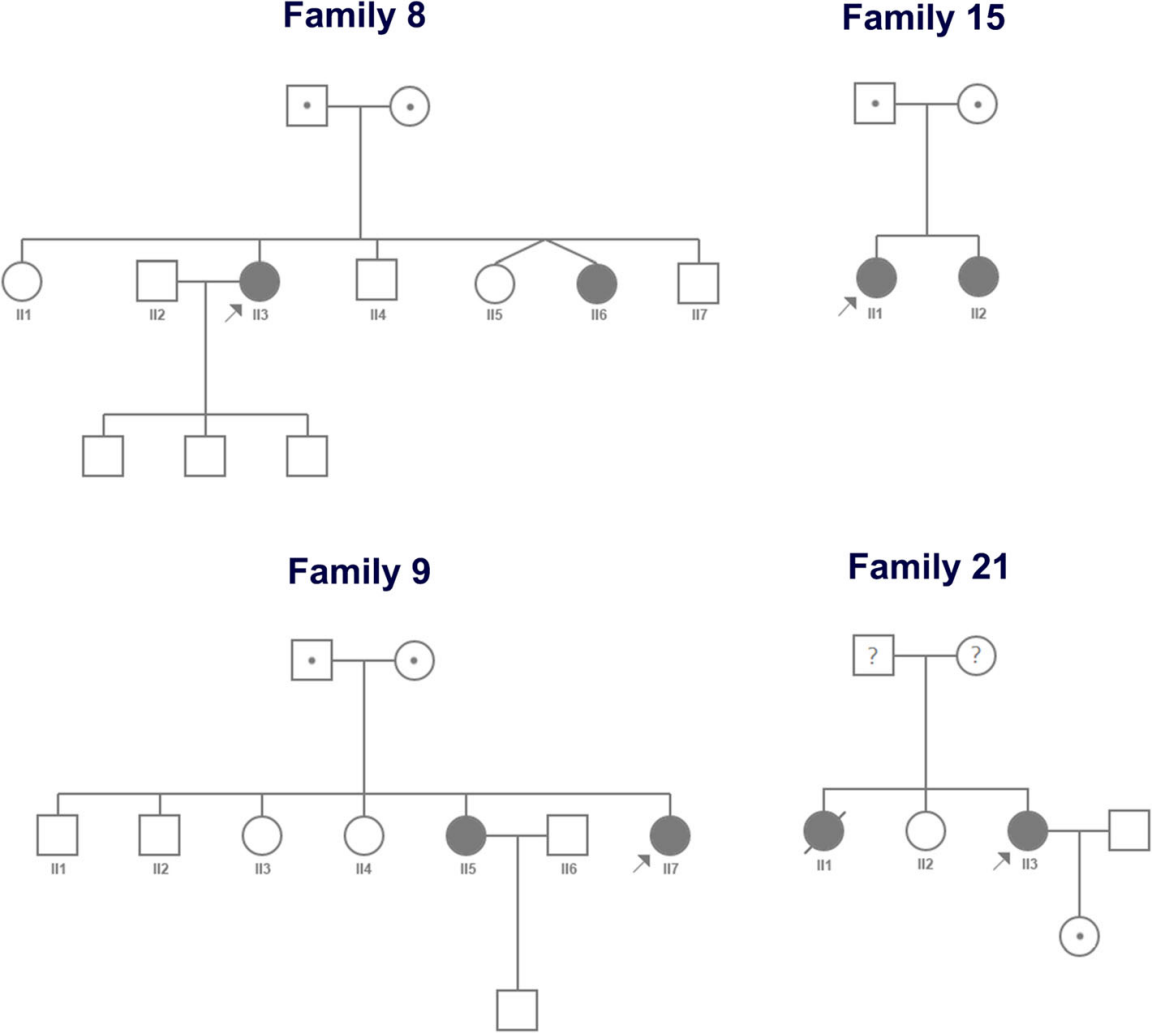
Pedigrees of families with several patients

Homozygous variants were found in probands 1, 3, 4, 7, 17, and 23, and definite consanguineous parents were determined in families 1, 3, and 7 (pedigrees shown in Fig. 2). In addition, several patients were determined in families 1, 8, 9, 15, and 21 according to similar clinical manifestations (pedigrees shown in Figs. 2, 3). The brother of proband 1 (IV5; Fig. 2) and the sister of proband 15 (II2; Fig. 3) had the same variants as the probands. The late brother of proband 1 (IV7), the sister of proband 8 (II6), the sister of proband 9 (II5), and the late sister of proband 21 (II1) were not analyzed by genetic testing (Figs. 2, 3). Other pedigrees are shown in Additional file 2.

De novo variants were determined in probands 18, 19, 20, and 22. Probands 18, 19, and 20 were diagnosed with LGMD 1B with autosomal dominant inheritance. Interestingly, the variant of proband 22 with LGMD 2C (c.477delG (p.V160Lfs*8) could not be detected in his parents, which indicated the possibility of spontaneous mutation of this variant. Considering that DNA samples of parents were from the blood, we could not exclude the possibility of chimeras because the variants might exist in other organs, including the gonads.

## Discussion

In this study, we report a LGMD cohort in South China, wherein we identified deleterious variants in 23 out of 24 probands. Five clinical characteristics and three muscle MRI characteristics, which have rarely been reported in the relevant subtypes, were observed. NGS analysis allowed the detection of 18 novel variants, and to the best of our knowledge, Chinese LGMD 2C patients have never been reported in the English literature thus far.

### The most common LGMD subtypes in China are LGMD 2A, dysferlinopathy, sarcoglycanopathies, and LGMD 1B

Previous LGMD cohorts in North China and Northeast China reported the most common LGMD subtypes to be dysferlinopathy (49.52, 38.46%) and LGMD 2A (24.76, 46.15%), followed by sarcoglycanopathies (9.52, 7.69%) and LGMD 1B (6.67, 0%) [16, 44]. In the present cohort, these subtypes were also the top 4, but the relative frequency of sarcoglycanopathies was obviously higher (26.67%). Thus, dysferlinopathy is the most common LGMD subtype in the Chinese population, but the relative frequencies of sarcoglycanopathies and LGMD 2A differ between the southern and northern regions of China. Furthermore, although dysferlinopathy is also most frequent in Korean and Japanese populations, the frequencies of LGMD 2A, sarcoglycanopathies, and LGMD 1B vary among countries [32, 45]. Therefore, the top 4 LGMD subtypes in East Asia include dysferlinopathy, LGMD 2A, and LGMD 1B, but the ranks of LGMD 2A and LGMD 1B differ between regions. The frequency of sarcoglycanopathies varies greatly among regions, and the top 4 LGMD subtypes in Korea did not include sarcoglycanopathies [16, 32, 44, 45]. The reason for this phenomenon is not clear at present, which warrants further investigation.

### Rare clinical features in LGMD 2F, 2I, 1B, dysferlinopathy, and the early onset recessive Emery-Dreifuss-like phenotype without cardiomyopathy

Most of the patients included in this study presented with the classical symptoms of LGMD; however, five rare characteristics were observed, which were hypermobility of the joints in proband 11 with LGMD 2I, proband 17 with LGMD 2F, and proband 18 with LGMD 1B; polydactyly in proband 14 with dysferlinopathy; patent foramen ovale in proband 16 with dysferlinopathy; delayed development of language in proband 23 with LGMD 2F; and atypical features in proband 12 with the early onset recessive Emery-Dreifuss-like phenotype without cardiomyopathy.

Hypermobility of the joints in the upper limbs— observed in some patients as well, although rare for LGMD 1B, 2F, and 2I patients—is a distinctive feature of congenital muscular disorders [46], and it should be carefully considered when diagnosing LGMD. Furthermore, proband 14 with dysferlinopathy presented with polydactyly in both hands. Polydactyly-related genes should be sequenced further for investigating the correlation between dysferlinopathy and polydactyly in proband 14. Proband 16 with dysferlinopathy had patent foramen ovale, which has never been reported in this condition. Although recurrent fat embolic strokes in a patient with Duchenne muscular dystrophy (DMD) with long bone fractures and a patent foramen ovale have been reported [47], whether such a patient should be prioritized to undergo surgery to close the defect over patients with simple patent foramen ovale needs to be assessed further because there is no evidence that patients with dysferlinopathy are more prone to bone fractures. Furthermore, although delayed language development has been reported in sarcoglycanopathies, this aspect has not been reported in patients with LGMD 2F [48, 49]. Because patients with DMD can present with delay in achieving both language and motor milestones [50] and manifestations of patients with sarcoglycanopathies are DMD-like [2], caution must be exercised to avoid the misdiagnosis of such patients with sarcoglycanopathies.

*TTN* gene variants were shown to cause titinopathy with various phenotypes, such as late-onset autosomal dominant tibial muscular dystrophy (MIM #600334), LGMD 2 J (MIM #608807), and hereditary myopathy with early respiratory failure (MIM #603689) [51]. Recently, a novel phenotype (early onset recessive Emery-Dreifuss-like phenotype without cardiomyopathy) was proposed, together with the description of three families with this phenotype, including one Algerian and two French Caucasian families [52]. Five main clinical features of this phenotype were proposed, including (1) the coexistence of both limb-girdle weakness and early-onset contractures, preceding or accompanying the initial weakness; (2) early-onset muscular dystrophy with a normal neonatal period, proximal weakness in infancy and childhood, and a progressive course in adolescence and adulthood, with permanent loss of ambulation from age 13 to 36 years; (3) unaffected facial, bulbar, and oculomotor muscles; (4) high CK levels, decreasing in the later stages of the disease; and (5) no identified cardiomyopathy to date. The clinical presentation of proband 12 with the *TTN* gene variants agreed with these reported features, even if his manifestations were relatively mild due to the early stage of disease; however, some variations were observed. In previous studies, early contractures were reported to be observed in limbs, especially the elbows and ankles, and only one patient was reported to have the contracture of the cervical spine at the advanced stage [52]. However, our patient was shown to have contractures of the neck and ankle rather than the elbows. In addition, the malformations of the feet were prominent, including cavus feet and morton’s toe in both feet. The CK level in this patient was less than twofold higher than the ULN, consistent with the mild weakness but unlike previously reported cases [52].

### Novel findings of muscle MRI features in LGMD 2A, dysferlinopathy, and the early onset recessive Emery-Dreifuss-like phenotype without cardiomyopathy

Among eight probands with muscle MRI, three had dysferlinopathy (probands 3, 5, and 13); two had LGMD 2D (probands 10 and 15); and one each had LGMD 2A (proband 9), LGMD 2I (proband 11), and the early onset recessive Emery-Dreifuss-like phenotype without cardiomyopathy (proband 12). The features of muscle involvements in probands 3, 10, 11, 13, and 15 were consistent with previous reports [53–57]. Although different degrees of fatty infiltration of the posterior muscle group of the leg can be observed at the early stage of LGMD 2I [55, 56], proband 11 at the earliest stage of LGMD 2I was characterized only by mild fatty infiltration in the adductor magnus, which has also been suggested in previous studies [57]. Thus, LGMD 2I should still be considered in such patients.

Furthermore, the features of fatty infiltration are atypical in probands 5, 9, and 12. Severe fatty infiltration of the thigh muscles observed in the MRI results obtained for probands 5 and 9 were consistent with severe motor dysfunctions as previously reported [55], but different muscle-sparing characteristics were observed in probands 5 and 9, who had advanced-stage LGMD. The MRI results obtained for proband 5, who had dysferlinopathy, showed that the adductor longus and gracilis were spared, unlike previously reported observations (the sartorius and gracilis were spared in advanced-stage dysferlinopathy) [13, 54], with the exception of a Chinese patient with dysferlinopathy reported to have similar muscle involvement [53]. In addition, the vastus lateralis, sartorius, and gracilis were shown to be commonly spared in the advanced stages of LGMD 2A, but proband 9 with LGMD 2A was shown to have the involvement of almost all muscles and a relatively mild involvement of the rectus femoris [55, 58]. Therefore, muscle involvement may differ between the populations, indicating the need for further muscle MRI analyses of Chinese patients with LGMD.

The clinical manifestation of proband 12 with the early onset recessive Emery-Dreifuss-like phenotype without cardiomyopathy has been discussed in the text above, and the muscle MRI results were also distinguished from those of a previous report [52]. Severe fatty infiltration of the semitendinosus and vastus lateralis with relative sparing of other muscles in proband 12 differed from previous findings of diffuse and severe fatty infiltration in the posterior group of thigh muscles or all thigh muscles [52]. These different MRI features might be correlated with the stage of disease, because MRI scans were tested 6 years and 13–17 years after the onset of symptoms in our patients and three previously reported patients, respectively [52]. Moreover, although an obvious weakness in ankle dorsiflexion muscles rather than ankle plantarflexion muscles could be observed, the anterior group of calf muscles (responsible for ankle dorsiflexion) was shown to be affected more mildly than those of the posterior group (responsible for ankle plantarflexion) according to MRI results, which may represent a characteristic of the early stage of the early onset recessive Emery-Dreifuss-like phenotype without cardiomyopathy.

### Genetic analyses

Our genetic analyses demonstrated that proband 6 has three variants in *DYSF* located in the same chromosome. The variant c.4742G > A with a minor allele frequency (MAF) of 0.003582 has been reported as a polymorphism in east Asia [59], while the variant c.565C > G was reported to disrupt the exonic splicing enhancer and affect RNA splicing [60], but it is relatively frequent in east Asia (MAF = 0.007787) and is considered benign/likely benign according to the ClinVar database [26]. This led to the reconsideration of the pathogenicity of c.565C > G and its classification as a SNP. A missense variant (c.5909C > T) with low MAF (not reported) was considered pathogenic. Patient 5 was found to carry two pathogenic variants in one chromosome (c.680 T > C; c.714G > T) and one pathogenic variant in this gene in another chromosome (c.799_800del). Furthermore, c.680 T > C was identified in a patient with dysferlinopathy [31], and both c.680 T > C and c.714G > T can be classified as pathogenic according to the ACMG guidelines [20]. Although it is unclear whether one of these variants is more pathogenic or not, it is not a crucial issue that will affect reproductive counselling, because, owing to their very small genetic distance, they are tightly linked [61].

The variant (c.545A > G) in *FKRP* has been reported to be a founder variant in a North China LGMD cohort [43], and the results of our study supported this theory. Besides, a LGMD 2I cohort from the Taiwan region indicated that half of the patients had the variant (c.545A > G) [39]. Thus, the variant (c.545A > G) might be a founder variant in China. The data of the variant spectrum of LGMD 2I in Korea and Japan are necessary to determine whether the variant (c.545A > G) was the hot spot variant in East Asia; this aspect warrants further investigation. Furthermore, proband 22 with LGMD 2C had compound heterozygous variants, one of which (c.756G > A) was inherited from his mother; however, another one (c.477delG) was not detected in his parents’ DNA samples extracted from blood cells. There are two possibilities to explain this phenomenon: (1) the de novo variant originated from spontaneous mutations during spermatogenesis or early development of the embryo, or (2) the father is a chimera: the variant does not exist in blood cells but might be present in other organs, including the gonads. If the father is a chimera with the variant (c. 477delG) in his testes, his future offspring would have a chance of being affected by the disease. As a next step, therefore, the father’s semen should be collected for gene sequencing to determine whether the variant exists in the gonads.

## Conclusions

In conclusion, this study described a LGMD cohort of 30 Chinese patients in 24 families carrying a total of 38 different variants. The frequencies of LGMD subtypes were discussed. Furthermore, five rare clinical characteristics and three rare muscle MRI characteristics were reported. These distinct clinical phenotypes and newly identified genotypes of patients in our LGMD cohort contributed to the novel recognitions of the clinical and genetic spectrums of LGMD, which may further promote a comprehensive understanding of the phenotype–genotype correlation and help improve the diagnosis and treatment of these diseases, as well as reproductive counselling.

## Additional files

Additional file 1: NGS protocol and the list of genes in the neuromuscular disorder panel. (DOCX 20 kb)

Additional file 2: Pedigrees of families with only one patient. (TIF 112 kb)

## Abbreviations

ACMG: American College of Medical Genetics and Genomics
CK: Creatine kinase
DMD: Duchenne muscular dystrophy
EMG: Electromyography
IHC: Immunohistochemical staining
LGMD: Limb-girdle muscular dystrophy
MAF: Minor allele frequency
MRC: Medical Research Council
MRI: Magnetic resonance imaging
NGS: Next-generation sequencing
SNP: Single nucleotide polymorphism
ULN: Upper normal limit
