# Supplementary material for: The clinical spectrum and genetic variability of limb-girdle muscular dystrophy in a cohort of Chinese patients
Source: Orphanet J Rare Dis. 2018 Aug 14;13:133. doi: 10.1186/s13023-018-0859-6 (PMC6092860; doi:10.1186/s13023-018-0859-6)
Supplement: Supplementary file 1 — NGS protocol and the list of genes in the neuromuscular disorder panel. (DOCX 20 kb) [file 13023_2018_859_MOESM1_ESM.docx]

1. **Target capture and sequencing**

Custom-designed NimbleGen SeqCap probes (Roche NimbleGen, Madison,Wis) were used for in-solution hybridization to enrich target sequences. Enriched DNA samples were indexed and sequenced on a Illumina sequencer (Illumina, SanDiego,Calif) with 150 cycles of paired end reads, according to the manufacturer’s protocols.

1. **Variant annotation and interpretation**

 Nucleotide changes observed of aligned reads were called and reviewed by using NextGENe software (SoftGenetics, State College, Pa). Besides detection of deleterious mutations and novel single nucleotide variants，coverage-based algorithm developed in-house, eCNVscan, was used to detect large exonic deletions and duplications.  The normalized coverage depth of each exon of a test sample is compared with the mean coverage of the same exon in the reference file, to detect copy number variants (CNVs).

1. **List of NGS neuromuscular disorder panel**

| SEPT9 | CHAT | EGR2 | HADHB | LITAF | PEX12 | PRPS1 | SYNE1 |
| --- | --- | --- | --- | --- | --- | --- | --- |
| AARS | CHKB | EMD | HOXD10 | LMNA | PEX13 | PRX | SYNE2 |
| ABHD5 | CHMP2B | ENO3 | HRAS | LPIN1 | PEX14 | PYGL | TARDBP |
| ACADM | CHRNA1 | ERBB3 | HSPB1 | LRSAM1 | PEX16 | PYGM | TCAP |
| ACADS | CHRNB1 | ERCC2 | HSPB3 | MED25 | PEX19 | RAB7A | TFG |
| ACADVL | CHRND | ERCC6 | HSPB8 | MFN2 | PEX2 | RAPSN | TNNI2 |
| ACTA1 | CHRNE | ETFA | HSPG2 | MPZ | PEX26 | REEP1 | TNNT1 |
| ACVR1 | CHRNG | ETFB | IGHMBP2 | MSTN | PEX3 | RIPK4 | TNNT3 |
| AGL | CLCN1 | ETFDH | IKBKAP | MTM1 | PEX5 | RYR1 | TPM2 |
| AGRN | CNTN1 | FBN2 | ISCU | MTMR14 | PEX6 | SBF2 | TPM3 |
| ALDOA | COL6A1 | FGD4 | ISPD | MTMR2 | PEX7 | SCN4A | TRIM32 |
| ALS2 | COL6A2 | FHL1 | ITGA7 | MUSK | PFKM | SCN4B | TRPV4 |
| ANG | COL6A3 | FIG4 | KBTBD13 | MYBPC1 | PFN1 | SCN5A | TTN |
| ANO5 | COLQ | FKRP | KCNA1 | MYBPC3 | PGAM2 | SEPN1 | UBA1 |
| AR | CPT1A | FKTN | KCNE1 | MYF6 | PGK1 | SETX | UBQLN2 |
| ARHGEF10 | CPT1C | FLNC | KCNE2 | MYH2 | PGM1 | SGCA | VAPB |
| ATL1 | CPT2 | FUS | KCNH2 | MYH3 | PHKA1 | SGCB | VCP |
| ATP2A1 | CRYAB | G6PC | KCNJ11 | MYH7 | PHKA2 | SGCD | VPS33B |
| ATP7A | CTDP1 | GAA | KCNJ12 | MYH8 | PHKB | SGCG | VRK1 |
| ATXN2 | DAG1 | GAN | KCNJ5 | MYOT | PHKG2 | SH3TC2 | WNK1 |
| BAG3 | DCTN1 | GARS | KCNQ1 | NDRG1 | PIP5K1C | SIGMAR1 | YARS |
| BIN1 | DES | GBE1 | KIF1A | NEB | PLEC1 | SLC12A6 | ZMPSTE24 |
| BSCL2 | DHCR24 | GDAP1 | KIF1B | NEFH | PLEKHG5 | SLC22A5 |  |
| C14orf133 | DMD | GFPT1 | LAMA2 | NEFL | PMP22 | SLC25A20 |  |
| C3orf39 | DNAJB6 | GJB1 | LAMB2 | NGF | PNPLA2 | SLC37A4 |  |
| CACNA1A | DNM2 | GLE1 | LAMP2 | OPTN | POMGNT1 | SMN1 |  |
| CACNA1S | DNMT1 | GYG1 | LARGE | PABPN1 | POMT1 | SMN2 |  |
| CAPN3 | DOK7 | GYS1 | LDB3 | PEX1 | POMT2 | SOD1 |  |
| CAV3 | DYNC1H1 | HADH | LDHA | PEX10 | PRKAG2 | SPTLC1 |  |
| CFL2 | DYSF | HADHA | LIFR | PEX11B | PRPH | SPTLC2 |  |
