# Supplementary figures and images for: The clinical spectrum and genetic variability of limb-girdle muscular dystrophy in a cohort of Chinese patients
Source: Orphanet J Rare Dis. 2018 Aug 14;13:133. doi: 10.1186/s13023-018-0859-6 (PMC6092860; doi:10.1186/s13023-018-0859-6)

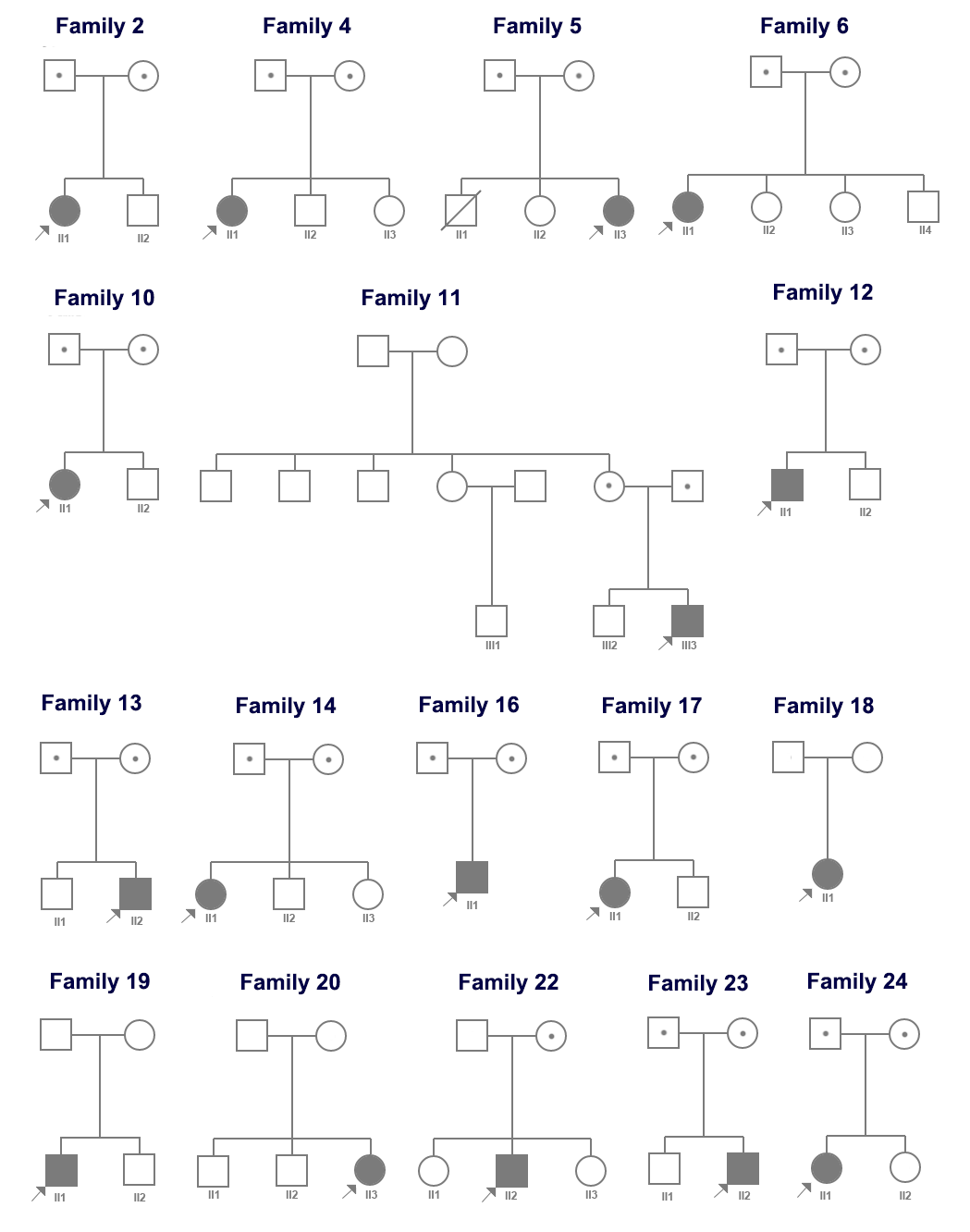

Supplement: Supplementary file 2 — Pedigrees of families with only one patient. (TIF 112 kb) [file 13023_2018_859_MOESM2_ESM.tif]
